# Blended learning in health education: three case studies

**DOI:** 10.1007/s40037-014-0108-1

**Published:** 2014-01-24

**Authors:** Nynke de Jong, Maggi Savin-Baden, Anne Marie Cunningham, Daniëlle M. L. Verstegen

**Affiliations:** 1 Department of Health Services Research, CAPHRI School for Public Health and Primary Care, Faculty of Health, Medicine and Life Sciences, Maastricht University, PO Box 616, 6200 MD Maastricht, the Netherlands; 2 Coventry University, Coventry, UK; 3 Institute of Primary Care and Public Health, Academic Lead for eLearning, School of Medicine, Cardiff University, Cardiff, UK; 4 Department of Educational Development and Research, Faculty of Health, Medicine and Life Sciences, Maastricht University, Maastricht, the Netherlands

**Keywords:** Blended learning, Synchronous online learning, Problem-based learning (PBL), Second Life learning, Web 2.0 technology

## Abstract

Blended learning in which online education is combined with face-to-face education is especially useful for (future) health care professionals who need to keep up-to-date. Blended learning can make learning more efficient, for instance by removing barriers of time and distance. In the past distance-based learning activities have often been associated with traditional delivery-based methods, individual learning and limited contact. The central question in this paper is: can blended learning be active and collaborative? Three cases of blended, active and collaborative learning are presented. In case 1 a virtual classroom is used to realize online problem-based learning (PBL). In case 2 PBL cases are presented in Second Life, a 3D immersive virtual world. In case 3 discussion forums, blogs and wikis were used. In all cases face-to-face meetings were also organized. Evaluation results of the three cases clearly show that active, collaborative learning at a distance is possible. Blended learning enables the use of novel instructional methods and student-centred education. The three cases employ different educational methods, thus illustrating diverse possibilities and a variety of learning activities in blended learning. Interaction and communication rules, the role of the teacher, careful selection of collaboration tools and technical preparation should be considered when designing and implementing blended learning.

## Introduction

The potential for reaching learners around the world increased greatly with the advent of the Internet and the World Wide Web. Rich educational resources are offered via online learning [1]. Some courses are completely online, others are blended learning courses. Blended learning was mainly found in higher education [2]. Technological developments, such as an electronic library and a virtual learning environment (VLE), have stimulated blended learning in higher education. Graham [2] claims that blended learning has grown rapidly and is predicted to become the ‘new traditional model’ or the ‘new normal’ in course delivery. What is meant by *blended learning*? Different dimensions of ‘blending’ were identified: blending instructional modalities, blending delivery media or blending instructional methods. The term blended learning is sometimes also used to refer to the use of technology in face-to-face education, but in this paper we focus on blended learning as ‘*a combination of traditional face*-*to*-*face and online instruction*’ [2, p. 334]. The reason to conduct some learning activities online can differ. Online learning can be place and time independent [1], which can be attractive for health care professionals who need to be lifelong learners in order to stay up-to-date [3]. When different countries are involved, learners not only acquire knowledge but also understand social and cultural traditions of different countries [4]. Cook [5] mentioned in particular disadvantages where the principles of effective learning were not incorporated into the initial programme design: social isolation, considerable up-front development costs, and occasional technical problems.

Novel instructional methods can be applied to individual modules, courses or programmes of study [6], for instance to make education more authentic and student-centred. It is generally accepted that curricula should be student-centred. Learning should be constructive, contextual, collaborative and self-directed [7]. These learning principles can be applied in the form of PBL and other similar approaches which can be characterized as guided learning in small groups that meet frequently. At first glance, this seems at odds with a blended learning approach in which a considerable part of the learning activities is executed online and at a distance.

From a technical viewpoint there are enough tools to communicate at a distance, both synchronously or a-synchronously. Examples of synchronous communication are chat, phone and web conference. Meetings are organized on fixed days and times. Students are communicating ‘*in* *real time*’. In a-synchronous communication, messages are posted by students at different points in time. Discussion forums, blogs, wikis and email messages are examples of a-synchronous communication. Most higher education institutes offer these communication tools via a VLE, such as Blackboard, FirstClass, or Moodle e.g. [3].

The central question in this article is: can blended learning be active and collaborative? This article does not pretend to summarize all the research in this area, but rather presents three case studies to answer this question. A sub-question is also formulated: which lessons can be drawn from the case studies?

In the first case study the existing successful PBL format was executed largely online but in essence is very similar to face-to-face PBL. In case 2 blended learning was introduced because the online activities in Second Life enable a learning activity that is potentially even more active and collaborative than would be possible face-to-face. In case 3 blended learning was meant to introduce collaborative learning in a situation where students previously worked fully individually.

Each case starts with a short description of the blended learning intervention followed by evaluation results. Space limitations make it impossible to describe the cases and evaluation methods in great detail. References are given to more elaborate reports.

## Casus 1: PBL in a blended learning format

### Background

This case describes the first course in a blended course in the Public Health Master’s Programme offered in English by the Faculty of Health, Medicine and Life Science at Maastricht University in the Netherlands. The university chose a blended (learning) format to accommodate working professionals following the Master’s programme part-time from a distance. The key advantage for students was the reduced travelling time [6]. The part-time group consisted of eight students, all working health care professionals. The tutor was a native English speaker.

### Blended learning design

**Fig. 1:**
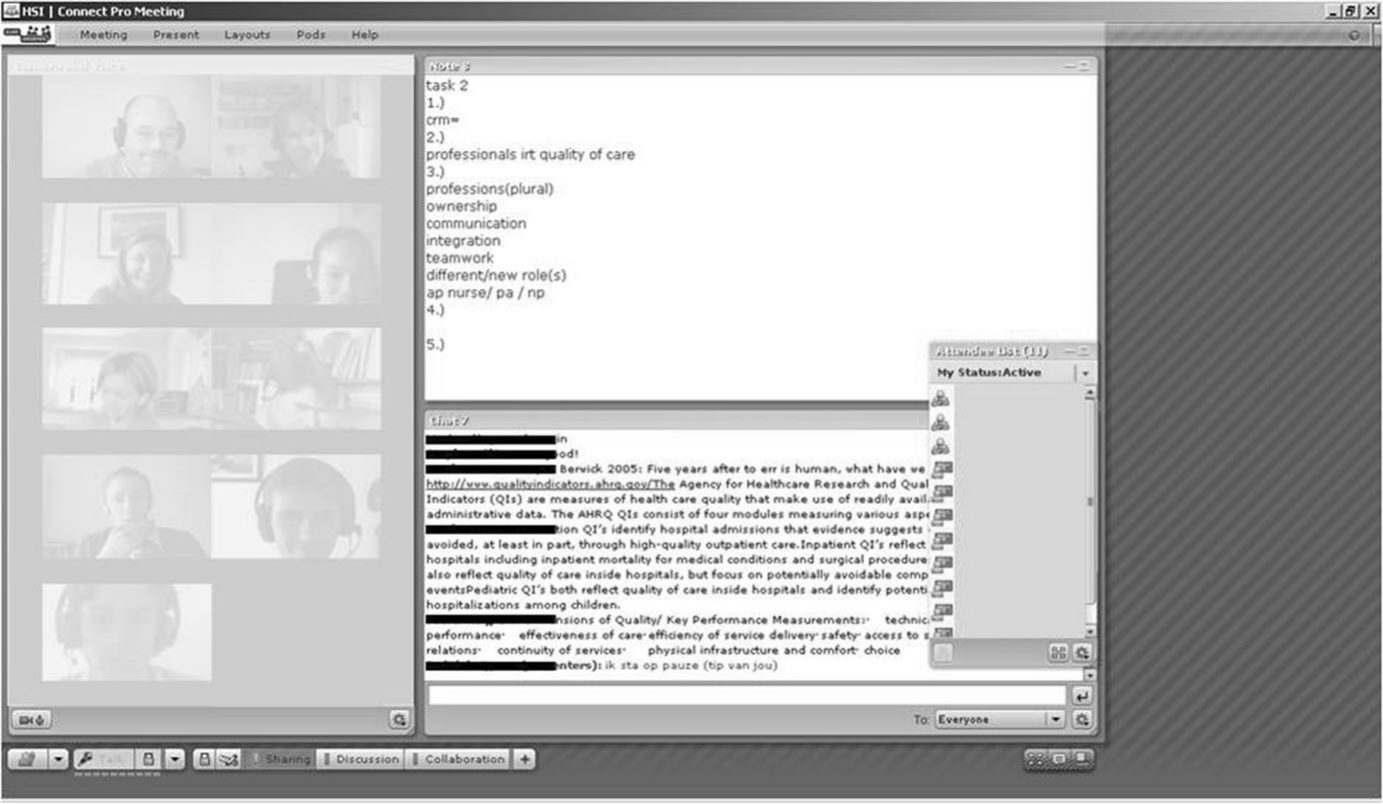
Preliminary discussion of a synchronous online PBL tutorial group meeting

Maastricht University has used PBL since the early 1970s. In traditional PBL, students work on tasks in small groups, using the seven-step approach. This approach can be divided into three parts: a preliminary group discussion, self-study and a reporting group discussion. In the synchronous online sessions the same approach was followed, but a virtual classroom was used for the synchronous online contacts (Fig. 1). All students needed an internet connection, headset and webcam.

In the preliminary discussion students brainstormed online about the problem and potential solutions based on their prior knowledge and they identified their own knowledge gaps. During the self-study period students worked individually, consulting scientific literature and other sources of information in order to find answers to the questions they had already defined. In the online reporting discussion students presented and discussed the newly acquired knowledge. Students had rotating roles in the online tutorials: each time one was discussion leader, one was scribe and the others were group members. They exchanged ideas, thoughts and views concerning a problem. A tutor supervised the online tutorial groups (cf. [8]).

There were two face-to-face meetings: an introduction day at the start (including the first tutorial group meeting and a dinner as social event) and in week 6 a knowledge test combined with a presentation of project plans by students. All other activities were online:

- 4 group meetings of 2 h on a fixed day in the week;
- 11 videotaped lectures;
- final presentation of the student projects.

### Evaluation

All students filled out a paper questionnaire (rated on a 5-point Likert scale) before and after the course (n = 8; response rate 100 %). Descriptive analyses were executed. In a focus group interview students were asked to discuss their experiences. The experiences were summarized. Results of the questionnaires were explained by using these experiences. The tutor was interviewed before and after the course. These interviews were also summarized.

The results of the study show that videotaped lectures and online discussions were acceptable to both students and tutor as a replacement for face-to-face activities. The overall quality of the course was labelled as good (Table 1). The content of the course, the PBL problems and the literature were judged as interesting and relevant. The social event at the beginning of the course seemed to have a positive influence on collaboration. Reduced travel time was seen as the main advantage. The online discussions were judged as equally or more effective and relevant than face-to-face discussions. Minor challenges were the lack of non-verbal communication cues and the fact that spontaneous remarks were difficult to make. In synchronous sessions rules for interaction and communication are important. Explicit turn taking is necessary to avoid interrupting each other. Careful selection of suitable tools and technical preparation and support were important. For students there was no difference in perceived tutor role. The tutor commented that it was difficult to deal with the extra task of managing the virtual classroom. The tutor’s attention was sometimes distracted from the discussion when technical problems occurred.

**Table 1:** Results with respect to the evaluation of the course

	Mean	n
The content of the course was interesting	4.3	8
The problems in the task were relevant	3.8	8
The literature was interesting	4.1	8
The quality of the course was good	3.8	8

Range is 1–5 (fully disagree–fully agree)

## Case 2: PBL in an immersive virtual world

### Background

Coventry University and St George’s Medical School in the United Kingdom have an innovative track record of using PBL since 1999. This case presents the PREVIEW project (PBL in Virtual Interactive Educational Worlds) that combined pedagogy with technology. The aims of the PREVIEW project were to develop and deliver PBL scenarios in an immersive virtual world (Second Life), to evaluate the scenarios from the users’ perspectives and to develop guidelines and best practice. The project was introduced to the part-time distance online MA in Health and Social Care Management at Coventry University and the second year of the three-year blended learning Paramedic Foundation Degree at St. George’s University of London and is now used in education, physiotherapy and psychology. In the first instance students at both universities were introduced to Second Life through a face-to-face induction session. The key advantages for staff and students were the flexibility, adaptability and realism offered by using PBL in an immersive world.

### Blended learning design

**Fig. 2:**
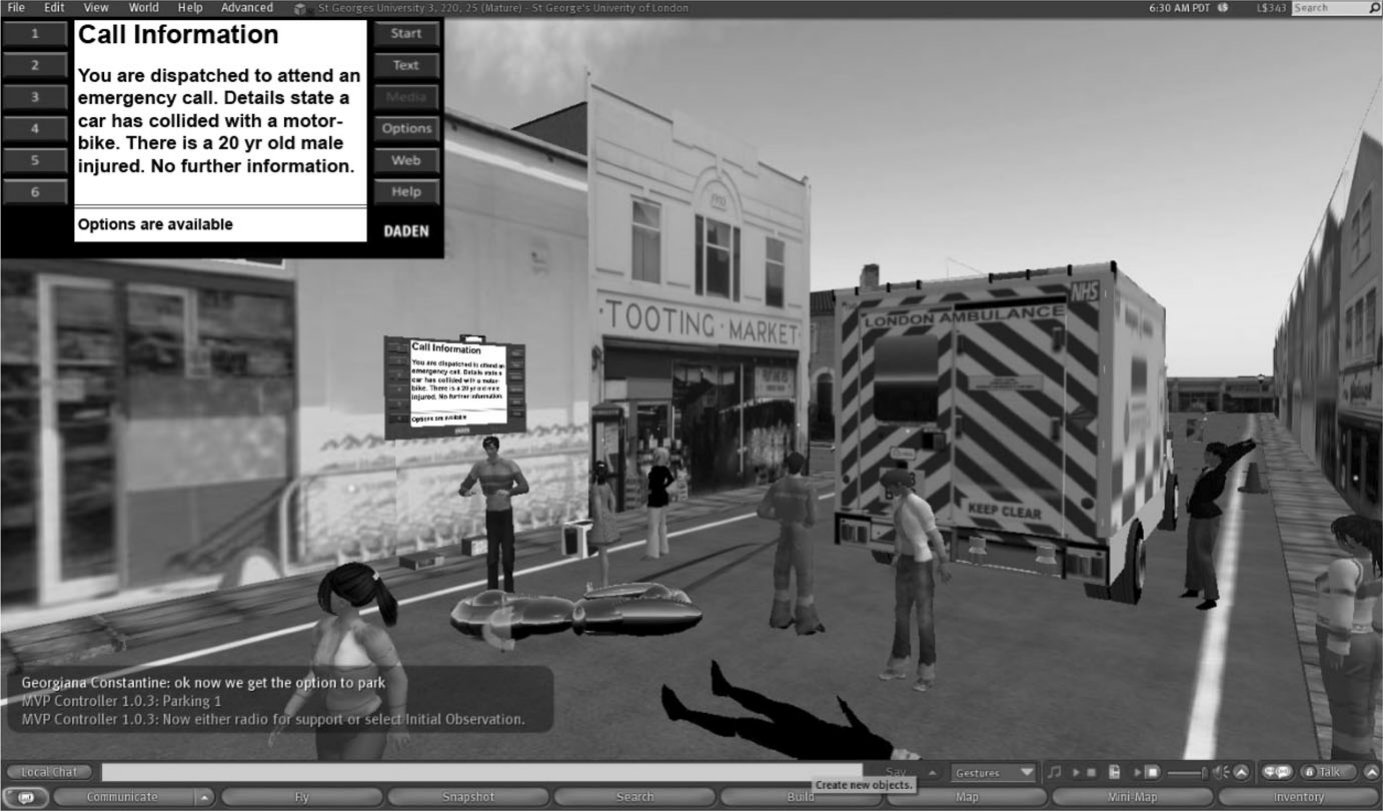
Second Life Street Accident Scenario with the patient mannequin on the floor near his motorbike

The PREVIEW project tested a replacement of traditional paper PBL cases with virtual patients delivered through a virtual world platform. The project team implemented and evaluated a user-focused approach to developing PBL environments and ‘good practice’ materials. This environment differs radically from the VLE in that it draws on a primarily visual set of semiotic resources with each participant having an online presence, or avatar, to aid their written communication, as seen in Fig. 2.

### Evaluation

Illuminative evaluation was used [9]. Data collection involved observation by an external evaluator and interviewing staff and students to explore and examine the interviewee’s perceptions. The objectives of the evaluation were to explore the impact of PBL in virtual worlds on learning and assess the usability of the learning environments. The result showed that:

- Second Life can provide a more authentic learning environment than classroom-based PBL and therefore changes the dynamic of facilitation. An immersive 3D environment can provide greater realism, active decision-making and a suitable environment for collaboration amongst work-based learners meeting.
- There are some technical considerations: relatively high specification computers and high bandwidth are required, and the interface is not as intuitive as might be hoped.
- Familiarity with the environment improved usability. Initially, interaction with Second Life objects was not intuitive and labelling of widgets/objects within the scenarios was needed.
- It is essential to prepare users through structured, context-related orientation sessions prior to use.
- Some barriers remain, such as time required to adjust to the platform, and development costs, but virtual worlds offered a unique opportunity to create realistic and immersive scenarios

An immersive 3D environment, such as Second Life, can provide an authentic learning environment [10]. Using PBL in Second Life embraced issues such as student diversity and improving student engagement [11] connected with complex curriculum design and the need for complex PBL scenarios to be developed.

## Case 3: using Web 2.0 technologies in a VLE

### Background

The ‘Family Case Study’ is an example of early community-based clinical contact, designed to give students authentic early experience [12] which has been part of the medical course in Cardiff for more than 20 years [13]. All students in the second year of a 5-year course (approximately 300 students each year) visit a family over several months. This is supported by face-to-face teaching sessions to help students address learning outcomes and also to identify an aspect of the family’s experience to explore in greater depth.

### Blended learning design

Students visited the family in pairs and completed individual assessments. The aim was to encourage collaborative learning and, therefore, they were encouraged to share how they planned to address learning outcomes and to respond to each other’s queries within the forums. It was also important that they developed research skills in accessing a variety of content, including patient-generated materials such as posts in health communities and blog posts, as well as academic publications. They used this content when writing an essay which looked in depth at one experience of the family they were visiting.

**Fig. 3:**
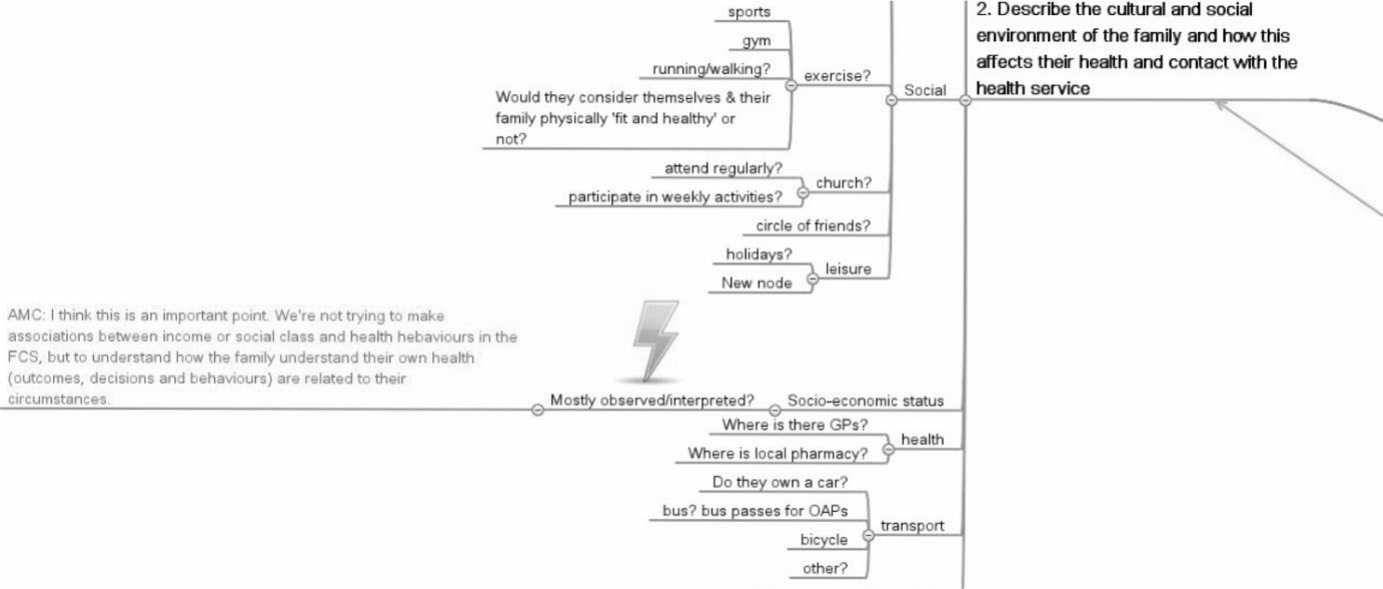
An example of a public editable mindmap (a ‘shared wikimap’) in case 3

Since this activity took place over an extended period with students organizing their own visits and study, it was appropriate to use asynchronous communication to provide support. In addition to lectures and small-group teaching sessions the university’s VLE was used, in particular a discussion forum, blogs and a wiki. This was supplemented with the use of social media and web 2.0 tools which were often embedded within the VLE. A public editable mindmap, (a ‘wikimap’) was used by students to share the approaches they were taking to address learning outcomes. Students were encouraged to ask the tutor questions about the project through the discussion forum, for example if they required assistance in identifying resources for their in-depth project. In response a short screencast could be produced to explain to students how to perform a good search. This was then embedded in the discussion forum. Identified resources were saved in social book marking tools and tag clouds were generated which again were embedded in the VLE. A Facebook page was created for the project and screencasts and resources were shared through this (Fig. 3).

### Evaluation

Students were surveyed in 2010 during and after the project to gain qualitative feedback on their experience of the use of these tools. The most popular and most used tool was the discussion forum. Some students saw the project as quite nebulous. Being able to access the questions of other students and tutor responses to these was highly valued as a way of bringing structure to their ideas. The wiki mind map was also highly accessed although less than 5 % of the students engaged in editing it. Some students reported being overwhelmed by the variety of resources at an early stage in the year. One student said ‘I feel there are too many new programmes to familiarize with’ and another said, ‘I feel a bit lost in a sea of material’. Another student described a worry about getting left behind: ‘Worried that as a medical student I’m not that technologically competent; I’m not a complete technophobe but talks of blogs and diigo and discussion forums really confuses me and I would have no idea about how to use it and therefore I’m worried that I’m going to miss something important which will be detrimental to my essay.’ The use of screencasts to help students with search strategies was particularly mentioned in the final feedback because it helped students to access references of an appropriate level.

In summary, the use of social media tools to supplement those available in the VLE can aid students, and encourage interaction with staff and other students. Clear explanation is needed to avoid students feeling burdened by new tools.

## Conclusions and recommendations

Blended learning makes it possible to take advantage of the opportunities of both online and face-to-face learning. But can blended learning be active and collaborative? This is the central question of this paper. The three cases discussed above clearly show that active collaborative student learning is also possible at a distance. Online learning activities can be efficient and attractive. Innovative educational formats are accessible and manageable from everywhere in the world. The sub-question of this paper ‘*Which lessons can be drawn from the case studies*?’ is answered below. Face-to-face activities may be necessary for learning activities that cannot take place online (e.g. skills practice), but they also make sure that students and teachers know each other, which makes it easier to collaborate online as well. In situations where online activities dominate introducing a social event at the start may be a good idea [14]. Donnelly [15] reported that the social context is critical for the success of a course. In online learning the role of the teacher might change. This was, for instance, visible in case 1 where the tutor had to deal with the double task of supervising the discussion and managing the virtual classroom. Moreover, tutors should be aware that online behaviour is difficult to interpret due to the invisibility of nonverbal cues [15].

All three cases show the necessity of careful selection of tools and planning, preparation and support in the technical domain [14].

The three cases differ from each other, thus illustrating possibilities in blended learning. There is no standard solution: the optimal mix of learning activities in blended learning depends on the content and learning objectives, the target group of participants and the facilities available [14]. It should be noted that distance learning offers a challenge to think about educational formats and resources. A ‘one size fits all’ model does not exist [16]. The reasons to go blended also play a role. In case 1, reducing travel time for part-time students was a main goal. The results show that there was no loss of quality: online discussions and video lectures were equally effective and more efficient for students. In case 2 adaptability and realism were important goals. The results show that students can be involved in authentic tasks in an immersive, virtual world. An interesting consequence of the richness and authenticity of the Second Life scenarios is the large amount of detail provided, much more than is usual in paper-based face-to face PBL sessions. Second Life can provide a more authentic learner environment than classroom-based PBL and therefore changes the dynamic of facilitation, but at this stage it is not clear how this impacts on the way the scenario is used and facilitated. The goal of case 3 was to give students (authentic) early experience of community-based clinical contact. The use of social media tools and other tools available in the VLE helped students and encouraged interaction with staff and fellow-students. The availability of online tools is not in itself a good enough reason to use them. The way they are applied should be underpinned by knowledge about how people learn and respond to online learning tools [17].

Synchronous online communication was used in two cases described here (case 1 and 2). An advantage is that teachers have some control over the participation and time investment of students: students *must* show up in the virtual environment at a certain time. This can motivate students to spend time on studying and it can give teachers insight into what students have done. Another advantage is the possibility of giving instant feedback. Synchronous communication also has disadvantages. Especially for professionals working in healthcare it may be difficult to get time off work at a specific moment. Asynchronous communication is more flexible and may also be more suitable for topics that require careful reflection or further study [14]. In case 3 asynchronous communication in the form of feedback through a discussion forum was highly valued for bringing structure to an open-ended project.

In both synchronous and asynchronous online communication agreement on interaction and communication rules is important, even more important than in face-to-face sessions [14]. In case 1 participants could see each other on webcams but the images were too small to see any non-verbal communication. In case 2 avatars were used by students and initially no verbal communication (although this was possible and used at a later date). It is, therefore, important to know how to behave in a virtual environment [14, 15]. What can you expect from each other? It is therefore recommended to explicitly address communication rules in a first session. When non-verbal communication is important a face-to-face activity is probably more suitable. Moreover, cultural factors need to be considered when designing a course [18].

Blended learning is an important medium for the future. The combination of face-to-face and online activities is attractive, for instance for postgraduate learners who need to be lifelong learners to keep up-to-date [14]. New knowledge and skills can be learned in an efficient way and transferred immediately. For undergraduate students blended learning is also interesting, for instance because it provides opportunities to involve experts from all over the world in their education or to collaborate with peers from elsewhere [14]. Blended learning may even make it easier to realize interprofessional collaborative learning.

### Essentials

- Blended learning makes it possible to take advantage of the opportunities of both online and face-to-face learning. The optimal mix of learning activities in blended learning depends on content and learning objectives, target group of participants and available facilities.
- Active, collaborative learning at a distance is possible. A variety of efficient, effective and attractive learning activities can be managed efficiently and effectively online. Face-to-face activities enable learning that cannot take place online (e.g. when non-verbal communication is important), but also makes sure that students and teachers know each other, which makes it easier to collaborate online as well.
- Careful selection of appropriate and manageable tools planning, and technical preparation and support are important.
- Interaction and communication rules and the role of the teacher in online sessions require extra attention.
